# A randomized controlled trial of bedside ultrasound RUSH process to improve the quality of anesthesia for elderly emergency surgery patients

**DOI:** 10.12669/pjms.38.6.5104

**Published:** 2022

**Authors:** Dawei Liu, Kun Chen, Yanfeng Yao, Jingfei Sun

**Affiliations:** 1 Dawei Liu, Department of Anesthesiology, Yongchuan Hospital of Chongqing Medical University, XuanHua Road 439, Chongqing, 402160, China; 2 Kun Chen, Department of Anesthesiology, Yongchuan Hospital of Chongqing Medical University, XuanHua Road 439, Chongqing, 402160, China; 3 Yanfeng Yao, Department of Ultrasound, Yongchuan Hospital of Chongqing Medical University, XuanHua Road 439, Chongqing, 402160, China; 4 Jingfei Sun, Department of Anesthesiology, Chongqing Yongchuan Dakang Hospital of Traditional Chinese Medicine, Phoenix Avenue No. 8, Chongqing, 402160, China

**Keywords:** RUSH process, elder patients, emergency surgery, intraoperative anesthesia management

## Abstract

**Objectives:**

The rapid ultrasound in shock examination (RUSH process) is an assessment of patient’s heart function, volume status, and vasculature, which can help anesthesiologist understand the patient’s physical condition. In this study, the RUSH process was applied to elderly emergency surgery patients to evaluate whether it is beneficial to maintain the patient’s vital signs stable during the operation.

**Methods:**

In this randomized controlled clinical study one hundred elderly patients who needed general anesthesia and emergency surgery from January 2021 to July 2021 were randomly divided into RUSH group (Group-A, n=52) and control group (Group-B, n=48). The main result include the area under the intraoperative blood pressure curve (AUC), liquid input, urine output, lactic acid levels, number of vasoactive drugs used.

**Results:**

There were no significant differences in patients’ basic information, preoperative blood pressure, intraoperative blood loss, intraoperative fluid input, intraoperative blood transfusion, and urine output. Intraoperative systolic blood pressure less than 90mmHg AUC of Group-A is less than Group-B(P<0.05), diastolic blood pressure less than 60mmHg AUC of Group-A is less than Group-B(P<0.05). After the operation, the blood gas analysis lactic acid level in Group-A was lower than that in Group-B(P<0.05). Group-A used more vasoactive drugs than Group-B(P<0.05).

**Conclusion:**

The bedside ultrasound RUSH process is of great significance for anesthesiologist to understand the preoperative physical condition of elderly emergency surgery patients, and is beneficial to maintain the stability of intraoperative vital signs.

## INTRODUCTION

Preoperative comprehensive assessment of the emergency surgery patients is a difficult challenge for anesthesiologists.^1^ Preoperative evaluation comprises many aspects, among which cardiac function assessment and volume assessment is the most important for anesthesiologists.^2^ Cardiac function and volume status have guiding significance for the treatment of critically ill patients. Appropriate treatment is based on a good understanding of the underlying pathophysiological mechanisms.^3^ Preoperative transthoracic echocardiography (TTE) performed by an anesthesiologist has been shown to change the cardiac diagnosis in 67% and the management plan in 44% of patients.^4^ The rapid ultrasound in shock examination (RUSH process) involves a 3-part bedside physiologic assessment simplified as the pump, the tank, and the pipes, and it has been developed specifically to address these key issues.^5^ After these three aspects of assessment, the anesthesiologist will have a comprehensive understanding of the basic condition of the patients. In our department, full examinations can be done in approximately 5 mins, for example a 4-chamber views can be achieved in only 10 seconds.^6^ Successful use of RUSH in the seriously ill patients has been published, making RUSH protocol more feasible.^5^,^7^

However, it is still unclear whether it can improve the quality of anesthesia for elderly emergency surgery patients and make the vital signs more stable during surgery. Therefore, this study intends to perform bedside RUSH assessment on elderly emergency patients to determine whether it can help anesthesiologists better understand the patient’s condition before surgery and maintain more stable intraoperative vital signs.

## METHODS

This study adopts a randomized controlled clinical study design and was registered in the Chinese clinical trial registry (registration No: ChiCTR2100042377), with the approval of the hospital ethics committee; (dated: July 6 2020), One hundred patients were included in our study in the period from January 2021 to July 2021 with informed consent of the patients and their families. The inclusion criteria were elderly patients older than 60 years old undergoing emergency surgery including orthopedics, general surgery, urology, thoracic surgery, neurosurgery, ASA grade III and above, regardless of gender. The exclusion criteria include patients with skin burns or infections in the ultrasound examination area, allergic to coupling agents, and patients who do not cooperate with follow-up, and patients with BMI greater than 28. The included patients were first stratified by the department to ensure an even number of patients in each department, and then randomized into RUSH group (Group-A) and control group (Group-B) (Fig.1).

**Figure:**
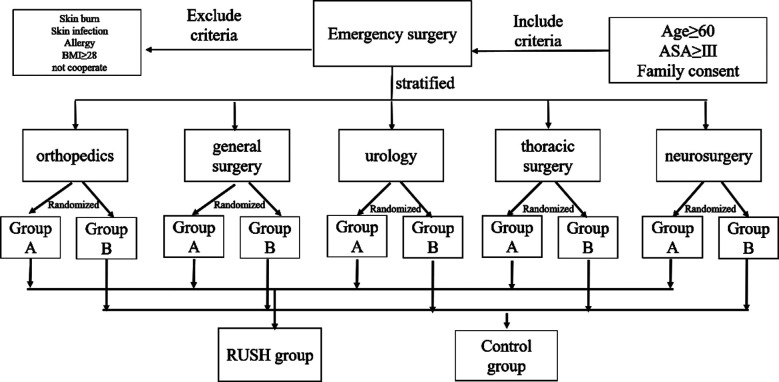
Fig.1

### RUSH process

The RUSH examination is performed immediately after admission using standard ultrasound equipment, we use a phased array probe 2.5-5MHz for cardiac ultrasound scanning and for the evaluation of pneumothorax, and a convex array probe 3.5-5MHz to allow abdominal scanning, and a linear array probe 7.5-10MHz for venous examination.^5^

The first step in the evaluation of the patient is determination of cardiac status, termed as the pump. Imaging of the heart usually involves four views include the parasternal long and short axis views, the subxiphoid view, and the apical four chamber view.^8^ we would get some measured value include ejection fraction (EF), interventricular septal thickness, left ventricular posterior wall thickness, mitral valve flow rate, aortic valve flow rate, and some relevant indicators when abnormal manifestations were found. Then we use this phased array transducer in the mid-clavicular line to identify the pleural line, A line is the normal appearance in the lung, B line represent the possibility of pulmonary edema, and the lung sliding motion can be depicted by using M-mode Doppler.^9^ when pneumothorax present, M-mode doppler will show only repeating horizontal line.

The second step is determination of effective intravascular volume status, termed as the tank. Placement of the probe in the subxiphoid position, get the long axis of the inferior vena cava (IVC) as it runs from the abdomen into the heart. The IVC is examined at the junction of the right atrium and the cava, followed 2 to 3cm along the vessel. Respiratory dynamics of the IVC will provide an assessment of the patient’s volume status. A smaller caliber IVC<2cm diameter with an inspiratory collapse greater than 50% correlates to a CVP less than 5cm of water, and a larger sized IVC >2cm with the collapse less than 50% correlates to a CVP more than 10cm of water, in the other cases, the CVP is between 5-10cm.^10^ After IVC examination, we use the convex array probe to further assess the tank for abnormal leakiness. This examination includes the space between liver and kidney, the area around the spleen, and the area behind the bladder, also the ultrasound assists the evaluation of the thoracic cavity for free fluid.

The third step is the evaluation of the pipes, we focused more on the abdominal aorta and the deep veins of the lower limbs, the maximal diameter of the aorta measurement greater than 3cm is abnormal and defines an abdominal aneurysm, and the pathognomonic finding of deep venous thrombosis (DVT) will be incomplete compression of the anterior and posterior walls of the vein.^11^

At the same time of the evaluation, we fill the data into the ultrasonic record sheet, ultrasound diagnosis was made and anesthesia precautions were made.

### Anesthesia method

While in the emergency operation room, routine monitoring included electrocardiogram (ECG), pulse oxygen saturation (SpO2), noninvasive blood pressure (NIBP). Continuous invasive arterial blood pressure (ABP) was monitored through a catheter inserted into the radial artery. Anesthesia was induced by midazolam 0.1mg/kg, etomidate 0.15-0.2mg/kg, sufentanil citrate 0.3-0.4μg/kg, rocuronium bromide 0.6mg/kg. Endotracheal intubation was performed after three minutes oxygen mask inhalation. The ventilator parameters were set about volume 6-8ml/kg, respiratory rate 12 times/minutes, inspiratory/expiratory ratio 1:2. As for anesthesia maintain process, continuous inhalation of sevoflurane, intravenous pump remifentanil and propofol, application of rocuronium bromid intermittently, maintain the bispectral index (BIS) between 40-60. Vasoactive drugs and fluid transfusion were used according to intraoperative conditions. Muscle relaxants should be discontinued 30 minutes before the end of surgery. The patient should be resuscitated immediately or sent to ICU according to the condition of surgical site and the changes in vital signs during operation.

### Primary indicator

Area under dynamic blood pressure curve: Blood pressure of patients from entering the operation room to leaving the room was recorded. The DoCareVer5.0 (Medical system) was used to draw dynamic blood pressure curve. Systolic blood pressure above 140mmHg is defined as hypertension, and the area under the curve (AUC) above this blood pressure is calculated; Systolic blood pressure below 90mmHg is defined as hypotension, and the AUC below this blood pressure is calculated. Diastolic blood pressure above 90mmHg is defined as hypertension, and diastolic blood pressure below 60 is defined as hypotension, AUC is calculated in the similar way to evaluate the stability of the patients’ intraoperative circulation, the larger the AUC, the more unstable the vital signs.^12^

### Secondary indicators

The secondary indicators include fluid consumption, urine output, lactic acid, frequency of vasoactive drugs used, number of transfer to ICU, and number of death at 28 days after surgery.

### Statistical Analysis

SPSS 26 statistical software was used for statistical analysis, α was set to 0.05, and bilateral test was used for testing. For measurement data conforming to the normal distribution, the t test was used for comparison between groups, the Mann Whitney u test was used for the comparison between the non-normal distribution data, and the repeated measurement analysis of variance was used for repeated measurement indicators. The count data, number of transfer to ICU and number of death at 28 day after surgery between the two groups were compared using the χ2 test, and the Fisher exact probability method was used for those that did not meet the requirements of the χ2 test. Rank sum test was used for grade data.

## RESULTS

A total of 105 patients were enrolled in this study, five of these patents were eventually lost to follow up and excluded. The patients were all distributed in different departments. There were no significant statistical differences in the ratio of male to female, age, BMI, ASA classification and preoperative complications between the two groups after statistical tests. (Table-I).

**Table-I:** Patients characteristics and clinical data.

Basic information	Group-A	Group-B
Total	52	48
Orthopedics	6	10
General surgery	9	6
Urology	6	5
Cardiothoracic	4	2
Neurosurgery	9	7
Gastrointestinal surgery	18	18
Male/Female	30/22	30/18
Age(year)	69.67±5.8	69.1±6.67
BMI	22.72±3.2	22.72±2.73
*ASA classification*		
III	35	33
IV	17	15
Hypertension	17	13
Diabetes mellitus	9	8
Coronary heart disease	5	5
Other complications	7	5

***Note:*** Data are shown as mean ± standard deviation ( *x̅* ±s) or as an actual number. Compared with Group-A, *p<0.05.

In Group-A, a total of 10 patients with valvular disease were found through the evaluation, including aortic regurgitation, mitral regurgitation, tricuspid regurgitation, and the degree of regurgitation was mild to moderate. Two cases of patients with a small amount of pericardial effusion were found. Most patients’ EF values were between 50%-70%. Assessing the central venous pressure by the width and variation of the IVC, we found that most patients’ CVP less than 10cm, of which 37 patients were less than 5cm, and 15 patients were between 5cm-10cm. In our study, the patient’s chest ultrasound was all A-line, and no B-line was found. In general, four patients with preoperative hypotension had hypovolemic shock and five had distributed shock (Table-II).

**Table-II:** Statistical scale for ultrasonic data.

Ultrasound data	Orthopedics	Urology	Cardiothoracic	Gastrointestinal surgery	General surgery	Neurosurgery
Valvular disease	2	1		4	2	1
Pericardial effusion				2	1	
EF(%)	61.50±5.36	60.50±4.37	63.50±5.69	59.58±5.78	62.50±5.20	60.56±4.13
50%-70%	6	6	4	17	9	9
<50%				1		
Coefficient variation of IVC(%)	52.17±9.54	56.5±10.29	70.75±18.64	52.33±16.94	66.14±13.10	64.33±12.82
CVP<5	3	5	3	11	7	8
CVP5-10	3	1	1	7	2	1
CVP>10						
Pleural effusion			2			
Ascites				2	2	
Pelvic effusion					1	
Pneumothorax			1			
A line	6	6	4	18	9	9
B line						
Thrombus of lower extremity veins				1		1
Hypovolemic shock			2		2	
Distributed shock				5		
Cardiogenic shock						
Obstructive shock						

There was no statistically significant difference in the basic blood pressure of the two groups(P>0.05). Comparing AUC of systolic blood pressure greater than 140mmHg had no statistical difference(P>0.05), while the AUC of systolic blood pressure less than 90mmHg was significantly greater in Group-B than in Group-A(P<0.05). The AUC of diastolic blood pressure greater than 90mmHg was not statistically different(P>0.05), while the AUC of diastolic blood pressure less than 60mmHg was significantly larger in Group-B than in Group-A(P<0.05). The comparison of other indicators of intraoperative blood loss, crystal input, colloid input, blood transfusion, and urine output showed no statistical difference between the two groups(P>0.05). Compared with Group-A, the level of lactate after operation in Group-B was significantly higher(P<0.05). The number of patients in Group-A used more vasoactive drugs than Group-B(P<0.05). However, there was no significant difference in the number of transfer to ICU and the number of death at 28-day after surgery between the two groups(P>0.05) (Table-III).

**Table-III:** Comparison of intraoperative and postoperative indexes between the two groups

	A group(n=52)	B group(n=48)	T	P
Systolic pressure(mmHg)	135.02±16.14	129.75±20.36	1.44	0.153
Diastolic pressure(mmHg)	77.63±11.09	76.46±14.31	0.457	0.649
Area under dynamic blood pressure curve		Z	P
systolic pressure>140mmHg	17.50(0.00,200.00)	0.00(0.00,400.00)	-0.54	0.589
systolic pressure<90 mmHg	0.00(0.00,0.00)	0.00(0.00,200.00)	-2.545	0.011*
diastolic pressure>90 mmHg	0.00(0.00,0.00)	0.00(0.00,0.00)	-0.554	0.579
diastolic pressure<60 mmHg	87.50(0.00,425.00)	350.00(55.00,1100.00)	-2.772	0.006*
intraoperative blood loss(ml)	40.00(20.00,175.00)	50.00(20.00,100.00)	-0.167	0.867
crystalloid solution(ml)	1000.00(500.00,1500.00)	1000.00(1000.00,1500.00)	-1.222	0.222
colloidal solution(ml)	500.00(500.00,500.00)	500.00(500.00,1000.00)	-1.148	0.251
RBC transfusion volume(ml)	0.00(0.00,0.00)	0.00(0.00,0.00)	-0.862	0.389
Plasma transfusion volume(ml)	0.00(0.00,0.00)	0.00(0.00,0.00)	-0.530	0.596
urine volume(ml)	300.00(200.00,400.00)	300.00(200.00,400.00)	-1.381	0.167
Lactic acid levels	0.650(0.500,0.825)	0.800(0.600,1.300)	-3.138	0.002*
			χ^2^	P
Number of Vasoactive drugs used	18	8	4.179	0.041*
Number of transfer to ICU	16	15	0.003	0.959
Number of death at 28 days after surgery	3	5	0.732	0.392

***Note:*** Compared with Group-A,

* p<0.05.

## DISCUSSION

The main finding of this study is that the bedside RUSH protocol assessment can help anesthesiologists better understand the emergency surgery patient’s condition and make the blood pressure more stable during operation, reduce the time and degree of hypotension, and improve the microcirculation perfusion of patients. These differences may affect the patient’s postoperative outcome and make the patient recover quickly after surgery and reduce postoperative complications.

Factors affecting blood pressure include heart rate, preload, myocardial contractility, and peripheral vascular resistance.^13^ There was a significant difference in the degree of stability of intraoperative blood pressure between the two groups, which may be due to ultrasound has guided the reasonable application of infusion and vasoactive drugs. The contraction of the left ventricle can be visually seen through the short-axis view of the left ventricle, determine stroke volume and evaluate EF by measuring left ventricular end diastolic volume and left ventricular end systolic volume. In our study evaluation, it was found that the majority of patients had EF between 50%-70%, which is a basically normal left ventricular systolic status. Assessing central venous pressure through inferior vena cava width and respiratory variability, and judging the patient’s cardiac preload, we found that the central venous pressure in most patients was within 10cm of water column.^14^,^15^ If the patient’s EF is low, it means that the patient’s cardiac systolic function is poor and myocardial contractility needs to be enhanced. If the patient has normal EF or high EF and blood volume is low, fluid rehydration therapy is required. If the patient’s blood volume and myocardial contractility are both good, but there is still hypotension, it may be that the patient’s peripheral vascular resistance is low, and vasoactive drugs are needed to increase the afterload.^3^ For blood pressure drop caused by different reasons, the RUSH process can quickly evaluated and accurately judged, making intraoperative fluid supplementation and vasoactive drugs more reasonable.^5^ In this study, there was significant difference in the application of vasoactive drugs, Group-A used more vasoactive drugs than Group-B, it means that ultrasound is easier to detect the causes of hypotension, vasoactive drugs can be timely applied to improve blood pressure stability, rather than simply increasing preload by fluid replacement.

Maintaining hemodynamic stability can ensure organ perfusion and tissue perfusion. Lactic acid is a metabolite of anaerobic glycolysis in the body.^16^ It can evaluate the patient’s systemic oxygen metabolism and tissue perfusion during surgery. If the lactic acid is greater than 2mmol/L, it means the patient has undergone a lot of anaerobic metabolisms, and its microcirculation perfusion is insufficient.^16^ In our study, the blood lactate level of Group-A was lower than Group-B, indicating that Group-A patients had better microcirculation perfusion during operation.

The stability of patient’s intraoperative blood pressure and the balance of organ perfusion are the results of the correct anesthesia decisions. Most of the evaluation of hemodynamics is through invasive arterial blood pressure, central venous pressure, pulmonary artery floating catheters, non-invasive cardiac output monitoring.^17^,^18^ It is difficult to meet the clinical requirements for rapid and stable evaluation in clinics.^18^ In our study, RUSH process was done within five minutes, we can roughly quantify the blood volume and systolic function by ventricular end-diastolic volume and end-systolic volume, and we can also get some other important information include the amount of pericardial effusion, pleural effusion and abdominal effusion, and venous thrombosis stability.^5^,^19^ Especially for trauma patients, if preoperative combined with undetected pneumothorax is very dangerous, after the induction of anesthesia, the patient’s breathing changes from spontaneous breathing to mechanical ventilation. Changes in the pressure in the chest cavity may cause aggravation of the pneumothorax and may contribute to formation of a tension pneumothorax,^20^ timely detection of pneumothorax before surgery is of great significance^21^. In this study, it was found that a patient had a pneumothorax, and a closed thoracic drainage was placed before the operation to avoid tension pneumothorax after induction of anesthesia. There was no significant statistical difference between the number of transfer to ICU and the number of death at 28 days after surgery. Although the Group-A was relatively low, the statistical difference was not reached, which may be caused by the relatively small sample size.

### Limitations of the study

It includes a small single-center clinical trial, and there are some uncontrollable factors likewise the preoperative examination of emergency surgery patients is not uniform enough. Although the patients were systematically evaluated by ultrasound before the operation, the patients were not continuously monitored by esophageal ultrasound during the operation. In the future, we will expand the sample size and conduct continuous esophageal ultrasound monitoring during the operation to determine whether the application of ultrasound in the perioperative period can improve the quality of postoperative recovery and improve the survival rate of patients.

## CONCLUSION

Studies have proved that bedside ultrasound applied by emergency physicians is of great help for rapid diagnosis and improvement of treatment plan for critically ill patients^22^, and our study has found that the anesthesiologist’s application of the bedside ultrasound RUSH process can provide elderly emergency patients with a comprehensive and rapid preoperative evaluation, allowing the anesthesiologist to better understand the patient’s capacity status, cardiac function status, and discover undiagnosed comorbidities before surgery and formulate an appropriate treatment plan.It is a technique that can be promoted clinically to provide a reference for anesthesiologists.

### Authors’ Contributions:

**JS:** Project design, data analysis, revised the manuscript, and is responsible for the integrity of the work

**DL:** Clinical anesthesia case collection, data analysis, and responsible for the accuracy of the work.

**KC:** Patients randomization and anesthesia case collection.

**YY:** Ultrasonic technical guidance and data analysis.
